# Description of the Novel Networking, Open Discussion, Engagement, and Self-Promotion (NODES) Framework for the Advancement of Women Physicians on Social Media

**DOI:** 10.2196/48965

**Published:** 2023-06-08

**Authors:** Tricia Pendergrast

**Affiliations:** 1 Northwestern University Feinberg School of Medicine Chicago, IL United States

**Keywords:** social media, gender equity, women in medicine, woman physician, NODES framework, self-promotion, networking, open discussion

## Abstract

The Networking, Open Discussion, Engagement, and Self-Promotion (NODES) framework is a strategy that women in medicine can deploy at conferences to broaden their professional networks and engage with colleagues. The NODES framework was designed and implemented for use at the Women in Medicine Summit, an annual conference that unites women to combat gender inequality in medicine. Intentional use of social media at conferences using the NODES framework by women in medicine can increase the visibility of research projects and may lead to speaking engagements and awards.

## Introduction

Women in medicine face many barriers to equity in compensation and academic promotion [1]. Social media applications such as Twitter lack the gatekeeping inherent to traditional academia and promote interactions with a larger community outside of one’s medical specialty or institution. Social media can be used to promote gender equity by bridging the gap in sponsorship and mentorship between women and men physicians, thwarting cultural taboos surrounding self-promotion by women, and increasing the visibility of women in medicine who are well-suited for speaking opportunities and professional awards [2-4].

Women physicians are less likely than men physicians to report that social media use expanded their research portfolio or resulted in a speaking engagement. This discrepancy is possibly explained by women researchers having fewer followers and lesser engagement on their social media accounts than their male peers [5].

Academic conferences strongly encourage the use of social media by attendees, organizers, and speakers. A substantial body of literature has documented the success of Twitter as a means to disseminate knowledge and facilitate discourse between experts and conference attendees [6]. Considering the popularity of social media at these events and the presence of a concentrated audience of professionals who share similar interests, conferences are the perfect venues for women physicians who want to increase engagement with their social media profiles and intentionally use social media to augment their professional advancement. This viewpoint article proposes and discusses the Networking, Open Discussion, Engagement, and Self-Promotion (NODES) framework, a novel strategy that women in medicine can deploy at conferences to broaden their professional networks and promote their research and scholarship.

## The NODES Framework

### Overview

The NODES Framework was designed and implemented for use at the Women in Medicine Summit, an annual conference that unites women from all medical disciplines to share evidence-based solutions and action plans to address gender inequality in medicine [7]. The Framework is presented annually to conference social media volunteers at the preconference summit. These volunteers have successfully used the NODES framework at the 2020, 2021, and 2022 Women in Medicine Summits to further their own professional goals and increase social media engagement with the conference and among its attendees.

### Networking

Increased mentorship of women physicians has been proposed as an intervention to address gender inequalities in medicine, especially as they relate to disparities in academic promotion [2]. Despite the importance of mentorship, women physicians are less likely than their male colleagues to have a mentor across various levels of seniority and struggle to access mentors at their own institution [8,9]. Networking is an expected and popular component of academic conferences that can facilitate novel professional relationships [10]. Women physicians can deploy social media as a networking tool at conferences to seek out mentors and connect with women in positions of leadership [9]. Successful networking can lead to a digital mentor-mentee relationship, which can lead to improved chances of obtaining research funding, increasing career satisfaction, and decreasing burnout [8].

Before deploying social media as a networking tool, users should ensure that their profile contains accurate personal and professional information. The biography (bio) of a social media account can be viewed as an electronic “elevator pitch” as it serves to introduce the user and provides a brief background story [8]. A bio should contain the user’s legal name and credentials (eg, “MD/MPH”), specialty, notable special projects (eg, multisite clinical research trials), and professional affiliations and activities (eg, serving as chair for a committee within the Society of General Internal Medicine). The relevant parties should be tagged within the bio, if applicable (eg, “@SocietyGIM DEI Chair”). If character limits allow, personal interests such as hobbies should be listed, as these are excellent casual conversation starters. Finally, while a professional headshot is not necessary for a social media account, the bio should contain a recent photograph with the user’s face clearly displayed.

**Figure 1:**
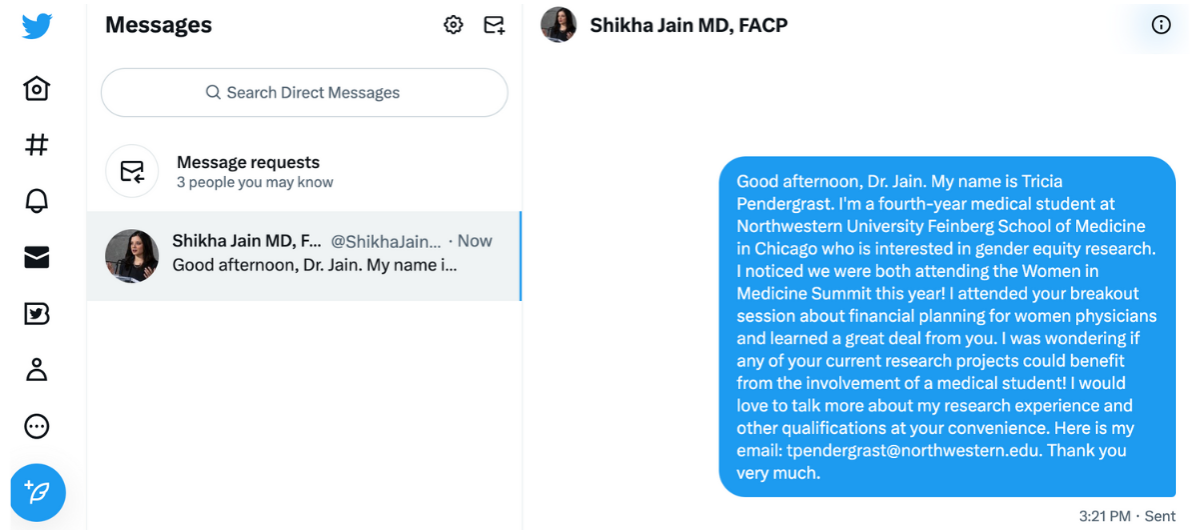
A screenshot demonstrating the act of networking using the direct message function on the social media application Twitter.

Cold calling is an accepted method of establishing a mentor-mentee relationship [8]. Networking on Twitter or Instagram should take place using the direct message function (Figure 1). The “cold call” message should begin with a salutation and an introduction that includes the sender’s name and correct title. The conference should be used as a connection (eg, “It looks like we were both attending the Women in Medicine Summit this year. I attended your financial planning session yesterday and really enjoyed it”). It is important that the message ends with one definitive request (eg, “I was wondering if any of your current projects could benefit from the involvement of a medical student”) and includes the necessary contact information to transition the conversation away from social media to more traditional forms of communication such as email.

### Open Discussion

Many women physicians experience difficulties with self-promotion. Cultural messaging leads women to view self-promotion as egocentric and unprofessional, which compounds existing inequalities for women in medicine [10]. It is recommended that women in medicine develop “strategic partnerships” with colleagues to promote each other’s accomplishments. These strategic partnerships should include *Open Discussion* (Table 1) of conference proceedings on social media. *Open Discussion* of research poster presentations, breakout sessions, plenary events, and oral presentations presented by women physicians may lead to subsequent research projects, speaking engagements, or awards [1]. *Open Discussion* can be deployed as a tool of solidarity by women colleagues or allyship by male colleagues on behalf of women physicians, who then benefit from increased exposure to their work [11]. By inviting the participation of male allies and other women colleagues, *Open Discussion* bypasses the challenges women in medicine face with self-promotion.

Social media users who plan to openly discuss research findings from a conference should ask permission of the presenter before doing so to prevent dissemination of confidential data. Appropriate attribution should be included in every *Open Discussion* post, which can be achieved by including the social media account of the presenter as well as tagging their institution and department. A link to any relevant research papers should be included in each *Open Discussion* post to increase the associated Altmetric score [1]. Photographs of the speaker, her slides (with permission), or other conference attendees can be included throughout *Open Discussion* posts. The inclusion of images increases the visibility of Tweets because images can be used to tag experts in the field, physicians with many followers on social media, and other conference attendees with Twitter accounts. These individuals receive a notification that they have been tagged in the post and can choose to comment or share it to their own accounts by “retweeting.”

**Table 1:** Categories and descriptions of open engagement with academic conferences on social media.

Category	Description
Notable fact	The social media user shares a key finding communicated by the presenter at a research conference, often using a citation or photograph of the poster or slide being presented.
Meaningful quote	The social media user shares an impactful quote communicated by a presenter, often during a plenary or presentation. Quotations are used and the phrase is appropriately attributed to its speaker.
Key takeaways	The social media user summarizes a presentation, research poster, oral presentation, or breakout session using bullet point phrases that contain the most important concepts.
Facts and figures	The social media user shares a table, figure, or statistics from a poster or oral presentation that demonstrates a key finding.

### Live Engagement

*Live Engagement*, colloquially referred to as “live-tweeting,” is a more time-intensive form of conference engagement than *Open Discussion* [12]. Similar to commentators on a televised broadcast of a sporting event, live engagement with programming at an academic conference functions much like a “play-by-play” of the action.

*Live Engagement* strategies contribute knowledge to nonattendees and function as a forum for discussion regarding the topics being presented [11]. Live-tweeting increases the visibility of the professional organization hosting the conference and communicates novel research findings to the broader scientific community and the public. Conference organizers may consider recruiting a team of volunteers to “live-tweet” certain key events, such as plenary presentations, or assign *Live Engagement* volunteers to presentations and sessions hosted by women physicians to amplify their work. These roles are an excellent way to engage medical trainees and younger faculty in service to a professional organization, which allows them to both attend the conference and add an instance of professional service to their curriculum vitae.

Prior to the event, *Live Engagement* volunteers should research the speaker and their work and attempt to identify important aspects of the session beforehand. Conference organizers may consider sharing a copy of the speaker’s presentation materials with *Live Engagement* volunteers in advance of the event. The quick pace of *Live Engagement* necessitates preparedness. Once the event commences, *Live Engagement* should occur in a linked “thread” of posts, which should all include the conference hashtag, the social media username of the conference’s sponsoring organization, and the speaker’s and their institution’s (and department’s) social media usernames, if applicable. Producing *Live Engagement* posts can be expedited by writing out the promoter’s username, her institution, and the conference hashtag in the initial Tweet and then copying and pasting this text into subsequent posts.

### Self-Promotion

Even though women in medicine face barriers to self-promotion, it is an essential component to social media engagement at academic conferences. These events attract a large, international group of people with shared academic interests and representation exists from every level of training, from medical student to the most senior faculty members.

In the context of an academic conference, *Self-Promotion* can take many forms. One tactic is to use social media to invite conference attendees to join a breakout session or seminar the social media user is leading while reflecting on the research paper or grant funding that was successfully obtained to facilitate the work being presented. Similarly, conference attendees can be invited to learn more about the speaker or her work by sharing an anecdote or tale of perseverance strategically tweeted out before or after an event featuring the social media user. Other examples of *Self-Promotion* strategies on social media include posts discussing current notable interspecialty or interinstitutional collaborations, posts about past speaking engagements, one’s research portfolio, and personal accomplishments such as successfully mastering a skill or having a child [5]. These topics invite other social media users to learn more about the woman behind the social media account as well as better understand her professional skill set for reference if future opportunities for collaboration arise.

Self-promoting posts not directly rated to a conference event should be scheduled to occur during breaks at the conference when attendees are more likely to check their phones or social media accounts. The visibility of these posts can be increased by including an image, tagging other conference attendees or colleagues, and using the hashtag associated with the conference.

## Conclusions

Social media applications offer women physicians networking and professional development opportunities. Despite this, women physicians are less likely than their male counterparts to use social media to expand their research portfolio [5]. The NODES framework can be used by women in medicine to increase engagement with their social media accounts at academic conferences, which may lead to the increased visibility of research as well as speaking engagements and awards. The strategies of including conference hashtags and tagging the usernames of other conference attendees should be used throughout the NODES framework to increase the visibility of posts. Future research should examine the impact of the NODES framework on research dissemination and opportunities for career advancement for women in medicine.

## Abbreviations

Bio: biography
NODES: Networking, Open Discussion, Engagement, and Self-Promotion
